# Identification of Candidate Male-Reproduction-Related Genes from the Testis and Androgenic Gland of *Macrobrachium nipponense*, Regulated by *PDHE1,* through Transcriptome Profiling Analysis

**DOI:** 10.3390/ijms25031940

**Published:** 2024-02-05

**Authors:** Shubo Jin, Yiwei Xiong, Wenyi Zhang, Hui Qiao, Yan Wu, Sufei Jiang, Hongtuo Fu

**Affiliations:** 1 Key Laboratory of Freshwater Fisheries and Germplasm Resources Utilization, Ministry of Agriculture and Rural Affairs, Freshwater Fisheries Research Center, Chinese Academy of Fishery Sciences, Wuxi 214081, China; xiongyw@ffrc.cn (Y.X.); zhangwy@ffrc.cn (W.Z.); qiaoh@ffrc.cn (H.Q.); wuyan@ffrc.cn (Y.W.); jiangsf@ffrc.cn (S.J.); 2 Wuxi Fisheries College, Nanjing Agricultural University, Wuxi 214081, China

**Keywords:** pyruvate dehydrogenase E1, testis, androgenic gland, RNA-Seq, male reproduction

## Abstract

The previous publication identified that pyruvate dehydrogenase E1 (*PDHE1*) positively regulated the process of male reproduction in *M. nipponense* through affecting the expressions of insulin-like androgenic gland hormone. The present study aimed to identify the potential male-reproduction-related genes that were regulated by *PDHE1* through performing the transcriptome profiling analysis in the testis and androgenic gland after the knockdown of the expressions of *PDHE1* by the injection of *dsPDHE1*. Both RNA-Seq and qPCR analysis identified the significant decreases in *PDHE1* expressions in the testis and androgenic gland in *dsPDHE1-*injected prawns compared to those in *dsGFP*-injected prawns, indicating the efficiency of *dsPDHE1* in the present study. Transcriptome profiling analysis identified 56 and 127 differentially expressed genes (DEGs) in the testis and androgenic gland, respectively. KEGG analysis revealed that the energy-metabolism-related pathways represented the main enriched metabolic pathways of DEGs in both the testis and androgenic gland, including pyruvate metabolism, the Citrate cycle (TCA cycle), Glycolysis/Gluconeogenesis, and the Glucagon signaling pathway. Thus, it is predicted that these metabolic pathways and the DEGs from these metabolic pathways regulated by *PDHE1* may be involved in the regulation of male reproduction in *M. nipponense*. Furthermore, four genes were found to be differentially expressed in both the testis and androgenic gland, of which ribosomal protein S3 was down-regulated and uncharacterized protein LOC113829596 was up-regulated in both the testis and androgenic gland in *dsPDHE1*-injected prawns. The present study provided valuable evidence for the establishment of an artificial technique to regulate the process of male reproduction in *M. nipponense*.

## 1. Introduction

*Macrobrachium nipponenese*, the oriental river prawn, is widely distributed through the whole of China [1]. It is the second culture freshwater prawn species in China. The annual production reached 220 thousand tons, producing huge economic benefits. The main culture regions in China include the Jiangsu, Jiangxi, Anhui, and Zhejiang provinces, where the annual productions are of over 20 thousand tons [2]. A previous publication has identified that both the testis and ovary of new hatchlings will mature within 45 days after hatching in the reproductive season. Rapid gonad maturation is the main problem, restricting the sustainable development of the *M. nipponense* industry. Rapid gonad maturation leads to inbreeding between the newborn prawns, resulting in having multiple generations in the same ponds, thus affecting the prawns in terms of market size [3]. Thus, it is urgently needed to establish an artificial technique to regulate the process of male reproduction in this species.

The glycolytic pathway is an important metabolic pathway to generate adenosine-triphosphate (ATP) in wide organisms. Glycolysis promotes the degradation and metabolism of glycogen or glucose under anaerobic conditions to produce ATP and is also a preparatory pathway for the aerobic oxidation of glucose in most organisms [4,5]. Pyruvate dehydrogenase (*PDH*) is an important enzyme involved in the glycolytic pathway. Glycolysis promotes the degradation of glucose to form pyruvate. *PDH* plays essential roles in the oxidation of glucose through catalyzing the oxidative decarboxylation of pyruvate to acetyl-CoA [6,7,8]. Furthermore, *PDH* has also been identified to link the glycolytic pathway with the tricarboxylic acid cycle (TCA cycle). Pyruvate dehydrogenase E1 (*PDHE1*) is a thiamine-diphosphate-dependent enzyme playing essential roles in catalyzing the oxidative decarboxylation of pyruvate. It is the rate-limiting step in the overall activity of *PDH* [9,10,11]. The absence of *PDHE1* has negative effects on the conversion of pyruvate into acetyl-CoA, thus resulting in a decrease in ATP production through the TCA cycle [12]. Previous studies have identified the significant morphological difference in the testis and androgenic gland between the reproductive season and non-reproductive season [13,14]. *PDHE1* was considered the candidate male-reproduction-related gene in *M. nipponense* because it was differentially expressed in the testis between the reproductive season and non-reproductive season [13]. *PDHE1* was further identified to positively regulate the process of male reproduction in *M. nipponense* through performing qPCR, in situ hybridization, and RNAi analyses. qPCR analysis revealed that *PDHE1* showed the highest expression level in the testis of *M. nipponense*. In situ hybridization analysis revealed that the signals of *PDHE1* mRNA were observed in the spermatogonia of the testis in *M. nipponense*. The injection of double-standard RNA of *PDHE1* (*dsPDHE1*) resulted in an over-95% decrease in *PDHE1* expression, and the decrease in *PDHE1* expression led to a 49% decrease in insulin-like androgenic gland hormone (*IAG*) expression. Histological observations revealed that less than 5% of cells were observed as sperm in the testis of *dsPDHE1*-injected prawns, which was significantly less than the percentage of sperm in the double-standard RNA of green fluorescent protein (*dsGFP*)-injected prawns (approximate 30%, in control). The knockdown of the expressions of *PDHE1* by RNAi can delay the testis reproduction in this species [15].

Androgenic gland is a specific organ that only exists in male crustaceans. It produces hormones that contribute to male differentiation and the development of male secondary characteristics in crustacean species, especially in the regulation of testis reproduction [16,17]. The ablation of the androgenic gland from male *Macrobrachium rosenbergii* has resulted in sex reversal to “neo-female” [18,19]. The androgenic gland has been observed in many crustacean species, and similar functions in the regulation of male reproduction have been reported. Insulin-like androgenic gland hormone (*IAG*) is the most focused gene secreted by the androgenic gland. *IAG* plays essential roles in the regulation of male differentiation and reproduction in many crustacean species [18,19,20,21]. A previous publication identified that the knockdown of the expressions of *IAG* resulted in sex reversal in *M. rosengergii* [22]. The testis has also been reported to regulate the process of reproduction, sexual maturity, and sex differentiation in crustacean species [23,24,25].

Many previous publications have focused on the process of male differentiation and reproduction in *M. nipponense*, using the androgenic gland and testis as the target tissues. The transcriptome of the androgenic gland and the cDNA library have been constructed in this species in order to select candidate male-reproduction-related genes [26,27]. Transcriptome profiling analyses of the androgenic gland and testis have been further conducted after the ablation of eyestalks from male prawns [28,29], as have comparisons between the reproductive season and non-reproductive season [13,14]. RNAi has been employed to analyze the functions of candidate male-reproduction-related genes in *M. nipponense*, and Succinate dehydrogenase complex iron sulfur subunit B [30], Cyclin A [31], Cyclin B [32], and Cyclin B3 [33] have been identified to positively regulate the process of male reproduction in the species. Thus, it is hypothesized that the genes regulated by the *PDHE1* in the testis and androgenic gland were predicted to be involved in the regulation of male reproduction in *M. nipponense*.

In the present study, we aimed to select the candidate male-reproduction-related genes regulated by *PDHE1* through performing transcriptome profiling analysis in the androgenic gland and testis after the knockdown of the expressions of *PDHE1* by RNAi. The present study will provide valuable evidence in the establishment of artificial techniques to regulate the process of male reproduction in *M. nipponense*.

## 2. Results

### 2.1. Measurement of the Efficiency of dsPDHE1

**Figure 1:**
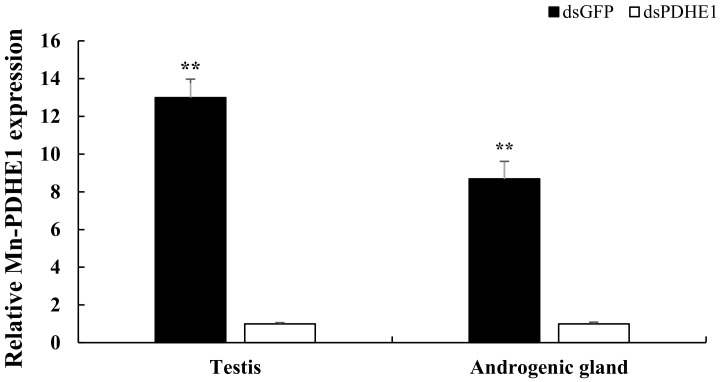
Verification of the efficiency of injected *dsPDHE1*. Data are shown as means ± SD (standard deviation) of tissues from three biological replicates. ** (*p* < 0.01) indicated the extremely significant difference of *Mn-PDHE1* expressions in the testis and androgenic gland between the *dsPDHE1*-injected prawns and *dsGFP*-injected prawns.

qPCR was used to verify the efficiency of *dsPDHE1* in the present study. qPCR analysis revealed that the expressions of *Mn-PDHE1* were significantly decreased in the testis and androgenic gland after the injection of *dsPDHE1*. The decreases reached 92.3% and 88.5% in the testis and androgenic gland after 5 days of *dsPDHE1* injection, respectively compared to those of *dsGFP*-injected prawns (Figure 1).

### 2.2. Transcriptome Profiling Analysis in Testis

**Figure 2:**
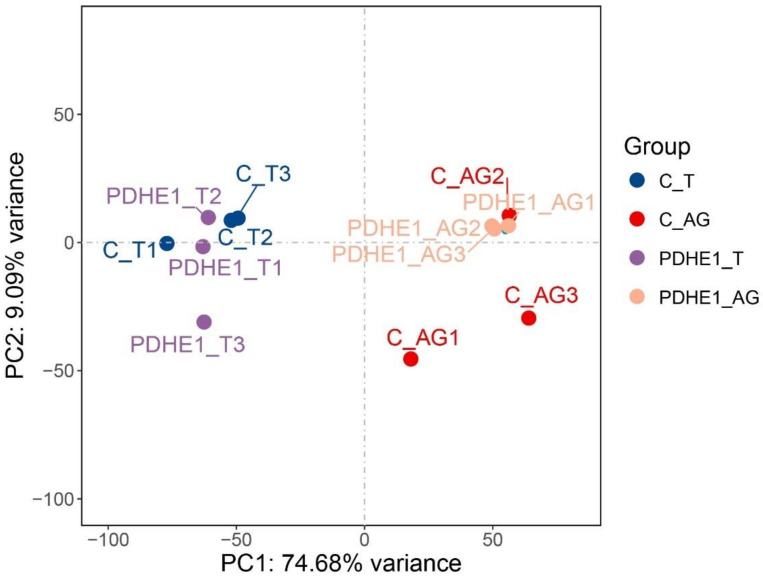
Principal component analysis for the testis and androgenic gland transcriptome profiling analysis using three biological replicates in *M. nipponense* after decreasing the expressions of PDHE1 by RNAi.

The principal component analysis was generated through the three biological replicates of the testis and androgenic gland transcriptome profiling analysis (Figure 2). Clear separations were observed between the samples of the testis and androgenic gland while the separations of the testis and androgenic gland between the dsPDHE1-infected prawns and dsGFP-injected prawns were not significant, indicating PDHE1 only regulated a limited number of gene expressions in the testis and androgenic gland, especially in the testis. In addition, PC1 and PC2 were responsible for 74.68% and 9.09% of variation sources, respectively, according to the extracted important variables.

**Figure 3:**
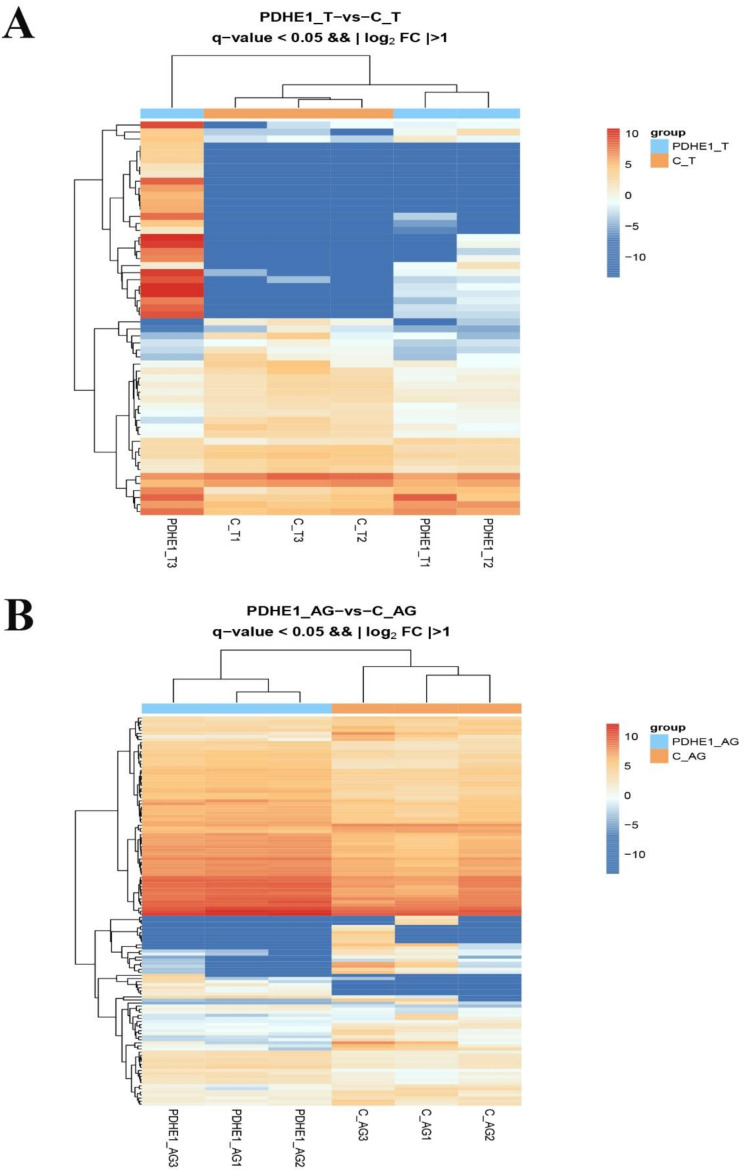
Clustering diagram of DEGs. The horizontal coordinates represent the sample names and the clustering results of the samples while the vertical coordinates represent the DEGs and the clustering results of the DEGs. The colors represent the expression levels of DEGs in the samples. (**A**) indicates the clustering diagram comparing the testis between the *dsPDHE1*-injected prawns and *dsGFP*-injected prawns. (**B**) indicates the clustering diagram comparing the androgenic gland between the *dsPDHE1*-injected prawns and *dsGFP*-injected prawns.

**Figure 4:**
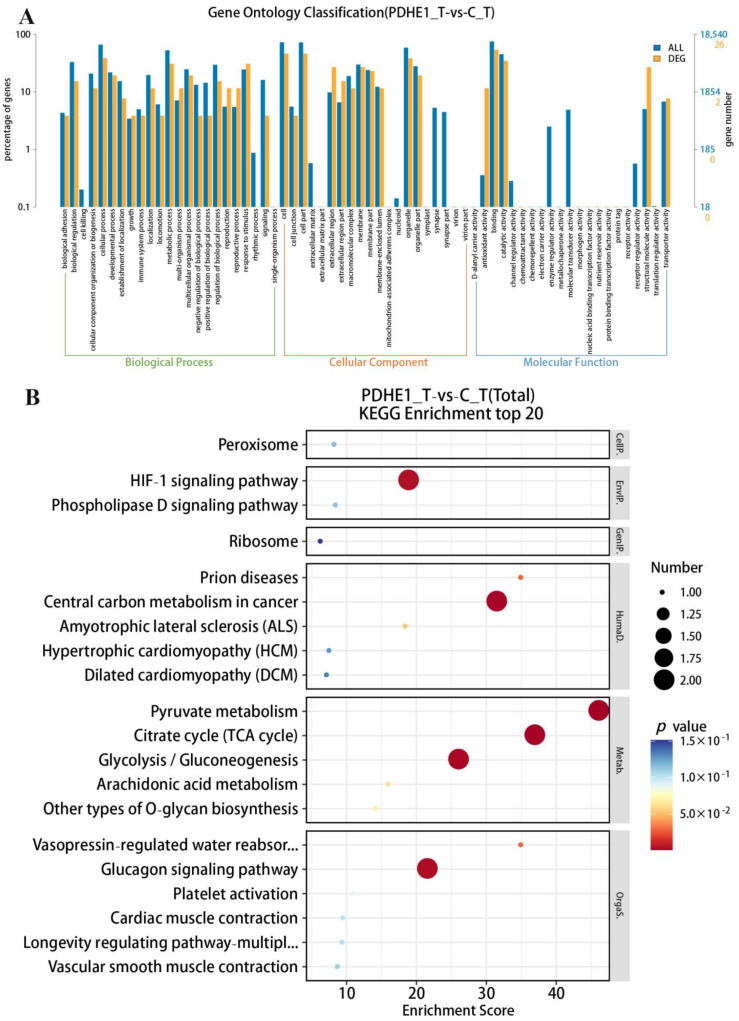
Gene Ontology (GO) analysis and Kyoto Encyclopedia of Genes and Genomes (KEGG) analysis of DEGs in the testis transcriptome after the injection of *dsPDHE1*. (**A**) indicates the GO analysis of DEGs. (**B**) indicates the KEGG analysis of DEGs.

A total of 20,053 genes were matched to the known genes of the *M. nipponense* genome in the testis transcriptome. The DEGs were selected through performing the transcriptome profiling analysis in testis after the injection of *dsPDHE1*, using the criterion of > 2.0 as being up-regulated and < 0.5 as being down-regulated. Fifty-six genes were differentially expressed after the injection of *dsPDHE1*, including thirty-three up-regulated genes and twenty-three down-regulated genes (Figure 3A). All of the DEGs can be seen in Supplementary Table S1. A total of 26 DEGs and 8 DEGs were annotated in the GO and KEGG databases, respectively. GO analysis identified 36 functional groups in the present study ranging from 1 to 14. Binding, Cell, Cell Part, Cellular Process, and Organelle represented the main functional groups of DEGs wherein the numbers of DEGs were > 10 (Figure 4A). KEGG analysis consisted of 25 metabolic pathways. Pyruvate metabolism, the Citrate cycle (TCA cycle), Glycolysis/Gluconeogenesis, the Glucagon signaling pathway, and the HIF-1 signaling pathway represented the main metabolic pathways of DEGs wherein the number of DEGs was 2. The other metabolic pathways only had one DEG (Figure 4B).

### 2.3. Transcriptome Profiling Analysis in Androgenic Gland

**Figure 5:**
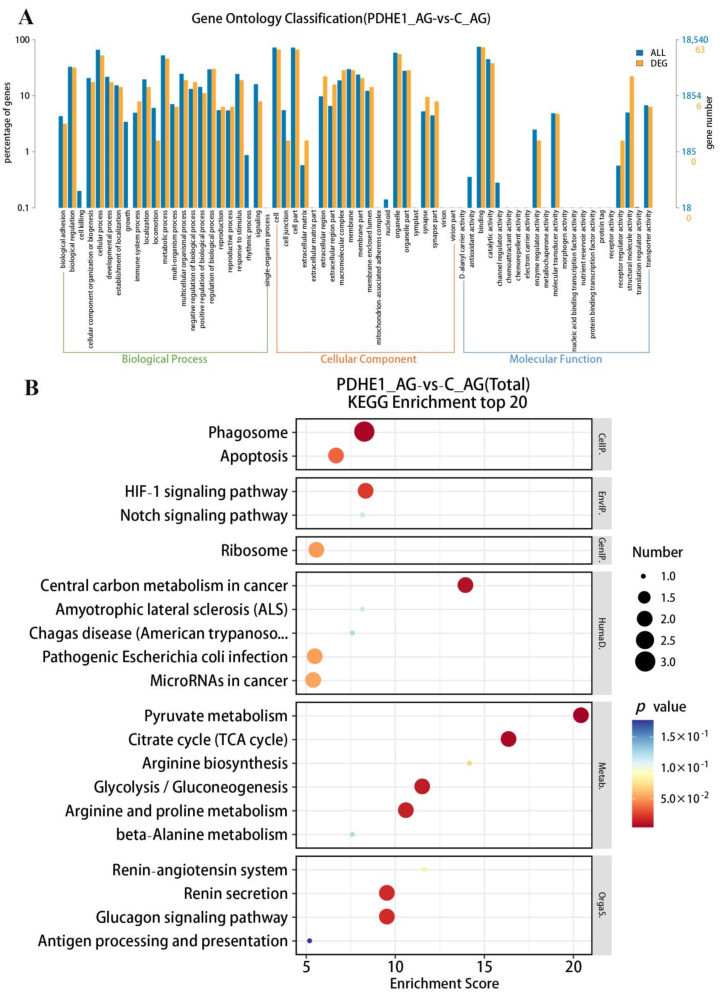
Gene Ontology (GO) analysis and Kyoto Encyclopedia of Genes and Genomes (KEGG) analysis of DEGs in the androgenic gland transcriptome after the injection of *dsPDHE1*. (**A**,**B**) indicate the GO analysis and KEGG analysis of DEGs, respectively.

A total of 17,035 genes were identified in the androgenic gland transcriptome that were highly matched to the known genes in the *M. nipponense* genome. A total 127 DEGs were selected in the androgenic gland after decreasing the expressions of *PDHE1*, including 76 up-regulated genes and 51 down-regulated genes (Figure 3B). All of the DEGs can be seen in Supplementary Table S2. A total of 63 DEGs and 18 DEGs were annotated in the GO and KEGG databases, respectively. A total of 40 functional groups were identified in the present study, according to the GO analysis. The number of DEGs in each functional group ranged from 1 to 46. Binding, Cell, Cell Part, Organelle, and Cellular Process represented the main functional groups of DEGs wherein the numbers of DEGs were > 30 (Figure 5A). KEGG analysis of DEGs identified 20 metabolic pathways ranging from 1 to 3. Phagosome represented the most enriched metabolic pathway of DEGs, and the other main metabolic pathways included pyruvate metabolism, the TCA cycle, Glycolysis/Gluconeogenesis, the Glucagon signaling pathway, the HIF-1 signaling pathway, Apoptosis, and Ribosome (Figure 5B).

### 2.4. Selection of Male-Reproduction-Related Genes

A total of 14 male-reproduction-related genes that were regulated by *PDHE1* were selected in the present study and considered as the strong candidate genes involved in the process of male reproduction in *M. nipponense*. Four genes were found to be differentially expressed in both the testis and androgenic gland, including calcification-associated peptide (*CAP*), chitin-binding-like protein (*CBP*), ribosomal protein S3 (*RPS3*), and uncharacterized protein LOC113829596 (*UP*). The other main down-regulated genes were selected from the testis and androgenic gland and listed in Table 1. These genes, which were positively regulated by *PDHE1*, were considered the candidate male-reproduction-related genes in *M. nipponense*.

### 2.5. qPCR Analysis of DEGs

**Figure 6:**
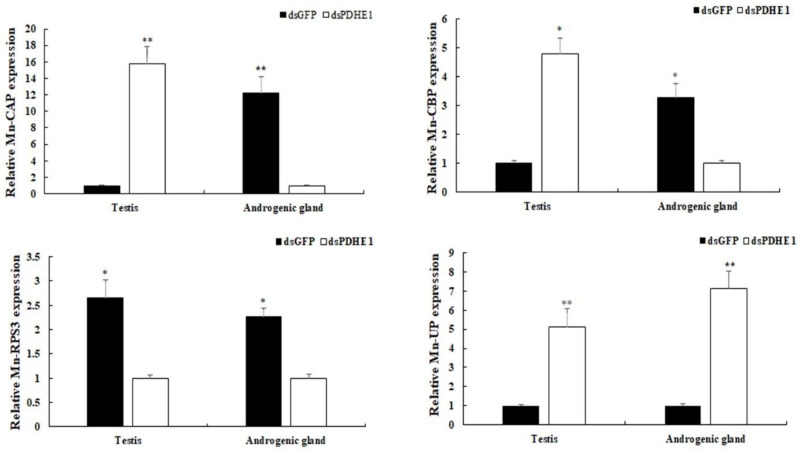
Verification of the expressions of DEGs in the testis and androgenic gland in the *dsPDHE1*-injected prawns and *dsGFP*-injected prawns. Data are shown as means ± SD (standard deviation) of tissues from three biological replicates. * (*p* < 0.05) and ** (*p* < 0.01) indicate significant difference and extremely significant difference in DEG expression between the testis and androgenic gland in the *dsPDHE1*-injected prawns and *dsGFP*-injected prawns.

The accuracy of RNA-Seq was verified by using qPCR analysis (Figure 6). The expressions of DEGs, which were differentially expressed in both the testis and androgenic gland, were selected to perform the qPCR analyses. The qPCR analyses generally showed the same expression patterns with those found via RNA-Seq. The expressions of *RPS3* were decreased by 62.3% and 55.7% in the testis and androgenic gland in *dsPDHE1*-injected prawns, respectively compared to those in *dsGFP*-injected prawns (*p* < 0.05) while *UP* expressions showed 512.3% and 713.5% increases in the testis and androgenic gland in *dsPDHE1*-injected prawns, respectively (*p* < 0.05). The expressions of *CAP* and *CBP* were increased in the testis in *dsPDHE1*-injected prawns while they were decreased in the androgenic gland in *dsPDHE1*-injected prawns (*p* < 0.05).

## 3. Materials and Methods

### 3.1. Sample Preparation

All of the prawns (*M. nipponense*) used for the present study were provided by Dapu *M. nipponense* Breeding Base in Wuxi, China (120°13′44″E, 31°28′ 22″N) in June 2022. All of the prawns were maintained in the lab condition for 3 days prior to the injection of *dsPDHE1* and *dsGFP*. The dissolved oxygen was > 6.0 mg/L.

### 3.2. Synthesize of dsPDHE1 and dsGFP

Previous publication has identified the specific RNAi primer with a T7 promoter site, which can efficiently knockdown the expressions of *PDHE1* in *M. nipponense* (Table 2) [15]. The *dsPDHE1* and *dsGFP* were synthesized by using the Transcript Aid™ T7 High Yield Transcription kit (Fermentas, Inc., Waltham, MA, USA) in the present study following the manufacturer’s protocol. *dsGFP* was used as the negative control [34].

### 3.3. RNAi Analysis

A total of 300 healthy male prawns with body weights of 3.74–4.58 g were used in the present study, which were randomly divided into *dsPDHE1*-injected group and *dsGFP*-injected group. Each group contained 150 prawns, and the groups were maintained in three separate tanks. Previous publications have identified the suitable injected dose of double-stand RNA as 4 μg/g in *M. nipponense* [30,31,32,33]. In the present study, the injected doses of *dsPDHE1* and *dsGFP* were both 4 μg/g, which was consistent with the previous studies. The *dsPDHE1* and *dsGFP* were injected into the prawns through cavum pericardiale. The injected prawns were maintained in the lab condition with the dissolved oxygen of > 6.0 mg/L. Snails were fed twice every day with a body weight of 2%. Both the testis and androgenic glands were collected at 5 days after the injection of *dsPDHE1* and *dsGFP*. Testis and androgenic gland samples were collected from fifteen individual prawns and pooled together to form a biological replicate for both transcriptome profiling analysis and qPCR analysis in order to provide sufficient tissues to extract RNA and eliminate the effects of individual differences. Three biological replicates were prepared for both transcriptome profiling analysis and qPCR analysis. The same biological replicates were used for the transcriptome profiling analysis and qPCR analysis.

### 3.4. qPCR Analysis

qPCR analysis was used to verify the efficiency of injected *dsPDHE1* and *dsGFP* in the present study, as well as for the verification of differentially expressed gene (DEG) expressions. RNA isolation and cDNA synthesis were performed according to the detailed description in the previous studies [30,31,32,33]. Briefly, total RNA was firstly extracted from each tissue sample using the UNlQ-10 Column Trizol Total RNA Isolation Kit (Sangon, Shanghai, China) according to the manufacturer’s protocol. The integrity of total RNA was measured by using 1.2% agarose gel electrophoresis, and an ultraviolet spectrophotometer (Eppendorf, Hamburg, Germany) was used to determine the RNA concentrations for each tissue sample. A total of 1 μg total RNA from each tissue sample was used to synthesize the cDNA template by using the PrimeScript™ RT Reagent Kit (Takara Bio, Kusatsu, Japan) according to the descriptions of the manufacturer’s protocol. The qPCR analysis was determined by using the UltraSYBR Mixture (CWBIO, Beijing, China), and performed on the Bio-Rad iCycler iQ5 Real-Time PCR System (Bio-Rad, Hercules, CA, USA). All qPCR reactions were run using three technical replicates. The primers for qPCR analysis were listed in Table 2, including the Eukaryotic translation initiation factor 5A (EIF), which was used as the reference gene in the present study. EIF has been identified as a suitable reference gene for PCR analysis in *M. nipponense* that can stably be expressed under various conditions [35]. The relative mRNA expressions were calculated using the 2^−∆∆CT^ method [36].

### 3.5. Transcriptome Profiling Analysis

The differentially expressed genes (DEGs) in the gills of *M. nipponense*, caused by the alkali treatment, were selected through performing the transcriptome profiling analysis. Previous publications have well described the detailed procedures for the RNA-Seq and analysis [13,14,27,28,29]. Briefly, the total RNA was extracted using RNAiso Plus Reagent (Takara Bio, Kusatsu, Japan) according to the manufacturer’s instructions. RNA integrity number (RIN) value of > 7.0 indicated the integrity of total RNA, measured by using A 2100 Bioanalyzer (Agilent Technologies, Inc., Santa Clara, CA, USA). RNA concentrations were measured using ultraviolet spectrophotometer (Eppendorf, Hamburg, Germany). A total of 4 µg of total RNA was used to construct the library. Illumina Hiseq-2500 sequencing platform was employed to perform the transcriptome profiling analysis under the parameter of PE150.

Fastp software (version 0.11.5) was employed to remove the low-quality raw reads with the default parameters [37]. The HISAT2 (version 2.2.1.0) software was then employed to map the obtained clean reads to the *M. nipponense* reference genome (Genbank access numbers: GCA_015110555.1 and GCA_015104395.1) [38]. Genes were annotated in the Gene Ontology (GO) (http://www.geneontology.org/, accessed on 15 August 2023) [39], Cluster of Orthologous Groups (COG) (http://www.ncbi.nlm.nih.gov/COG/, accessed on 15 August 2023) [40], and Kyoto Encyclopedia of Genes and Genomes (KEGG) databases (http://www.genome.jp/kegg/, accessed on 15 August 2023) [41], using FDR < 0.05. Gene expression was calculated using the FPKM method, where FPKM = cDNA fragments/mapped fragments (millions)/transcript length (kb), using HTSeq-count [42]. DESeq2 was used to perform the differential expression analysis [43]. The Benjamini–Hochberg correction method was used to calculate the false discovery rate (FDR) [44] with *q*-value < 0.05. Fold change > 2 was considered as marking up-regulated differentially expressed genes (DEGs), and fold change < 0.5 was considered as marking down-regulated DEGs.

### 3.6. Statistical Analysis

SPSS 23.0 was used to perform the statistical analyses in the present study, estimated using the Duncan’s multiple range test and LSD in one-way ANOVA. *p* < 0.05 and *p* < 0.01 indicated significant difference and extremely significant difference, respectively. Quantitative data were expressed as means ± SD.

## 4. Discussion

Rapid gonad maturation is the main problem affecting the sustainable development of the *M. nipponense* industry. Thus, the long-term goal of *M. nipponense* studies is aimed at establishing an artificial technique to regulate the process of gonad reproduction in this species. *PDHE1* has been identified to positively regulate the process of male reproduction in *M. nipponense* [15]. In the present study, the genes regulated by *PDHE1* were selected through performing transcriptome profiling analysis in the testis and androgenic glands of *M. nipponense* after the knockdown of the expressions of *PDHE1* by injecting *dsPDHE1*. The selected genes were considered as being the strong candidate genes involved in the regulation of male reproduction in *M. nipponense*, providing valuable evidence for the establishment of an artificial technique to regulate the process of testis reproduction in this species. The expressions of *PDHE1* decreased by 92.3% and 88.5% in the testis and androgenic gland in *dsPDHE1*-injected prawns after 5 days of *dsPDHE1* injection, respectively compared to those in *dsGFP*-injected prawns. In addition, the transcriptome profiling analysis also identified 88.1% and 87.3% decreases in *PDHE1* expression in the testis and androgenic gland in *dsPDHE1*-injected prawns, respectively, indicating that the injected *dsPDHE1* had efficiently decreased the expressions of *PDHE1* in the present study.

In the present study, a total of 56 and 127 DEGs were identified in the testis and androgenic gland, respectively according to the transcriptome profiling analyses after deceasing the expression of *PDHE1* by injecting *dsPDHE1*. KEGG analysis revealed that pyruvate metabolism, the Citrate cycle (TCA cycle), Glycolysis/Gluconeogenesis, the Glucagon signaling pathway, and the HIF-1 signaling pathway were identified as the main enriched metabolic pathways of DEGs in both the testis and androgenic gland. In addition, Phagosome, Apoptosis, and Ribosome were also identified in the androgenic gland.

Pyruvate is produced from the glucose metabolism through glycolysis. Thus, pyruvate metabolism plays essential roles for energy production and has been identified to be involved in various metabolic pathways, depending on the cell’s demands [45]. The TCA cycle and Glycolysis/Gluconeogenesis are essential metabolic pathways to produce energy in wide organisms [46]. Pyruvate is produced from glucose through the catalysis of Glycolysis/Gluconeogenesis, promoting the generation of ATP from free energy [47]. The glucagon signaling pathway is an important pathway for the body to regulate blood glucose levels under hypoglycemic conditions [48]. These metabolic pathways are energy-metabolism-related pathways, which have been predicted to be participated in the process of male reproduction through producing ATP in *M. nipponense* [13,14,28,29]. *PDHE1* is an energy-metabolism-related gene that has been identified to regulate the process of male reproduction in *M. nipponense* [15]. Thus, the DEGs from these metabolic pathways, which were regulated by *PDHE1*, were considered as the strong candidate genes that regulated the process of male reproduction by affecting the generation of ATP in *M. nipponense*. The potential functions of these DEGs in the process of male reproduction need further investigation.

Phagosomes are dynamic organelles formed by macrophages, playing essential roles in capturing and destroying microbial pathogens [49]. Apoptosis, which is regulated by a series of genes, is a process to capture and destroy damaged or aged cells in order to maintain a stable internal environment in order to better adapt to the living environment [50,51]. Ribosome exists in the cells of wild organisms. It promotes the process of correct translation from RNA to protein [52,53,54]. These metabolic pathways were also predicted to participate in the regulation of male reproduction through digesting the aged cells and ensuring corrected protein synthesis in our previous publications [13,14,28,29]. Thus, the DEGs from these metabolic pathways, which were regulated by *PDHE1*, were also predicted to regulate the process of male reproduction in *M. nipponense*.

Four DEGs were identified to be regulated by *PDHE1* in both the testis and androgenic gland in the present study, predicting that these genes were involved in the regulation of male reproduction in *M. nipponense*. *CAP* contains the repeat sequence of aspartic acid in the C-terminus, which plays essential roles for calcium-binding activity [55,56]. Previous publications have revealed that *CAP-1* is mainly involved in exoskeleton calcification in *P. clarkia* [55,56,57,58]. *CAP* has been also identified to play essential roles in the regulation of growth in crustacean species because it regulates the process of molting in crustaceans [59]. Chitin is mainly found in the exoskeletons of crustaceans and insects, playing essential roles in the stabilization of an arthropod’s body through acting as a physical defense against predators and desiccation [60]. Chitin biosynthesis plays essential roles in the regulation of the growth, development, and reproduction of insects and crustaceans [61]. The functions of *RPS3* are identified to be involved in the regulation of translation initiation [62,63], ribosomal maturation [64,65], the induction of apoptosis [66,67,68], the suppression of tumors [69], the maintenance of genomic integrity [70], DNA repair [71,72], and the regulation of the cell cycle and gene transcription [73,74,75]. *RPS3* is reported to be phosphorylated by cyclin-dependent kinase 2 (*Cdc2*) during the G2/M phase. *Cdc2* has been also identified to regulate the process of male reproduction in *M. nipponense* by RNAi [76]. The knockdown of the expression of *PDHE1* changes the process of male reproduction in *M. nipponense*. In the present study, the expressions of *RPS3* were positively regulated by *PDHE1*, predicting that it may regulate male reproduction through maintaining genomic integrity and ribosomal maturation. Uncharacterized protein LOC113829596 was also differentially expressed in both the testis and androgenic gland in the present study. However, its function is still unknown. The potential functions of this gene in the regulation of male reproduction need further investigation.

The main genes from the testis that were regulated by *PDHE1* included Glucoside xylosyltransferase 2 (*GXYLT2*), Cytochrome P450 3A (*CYP3A*), and Nesprin-1. These genes were also predicted to regulate the process of male reproduction in *M. nipponense*, based on the significant differences in gene expression between *dsPDHE1* prawns and *dsGFP* prawns. *GXYLT2* plays essential roles in the modification of the first xylose to the O-Glucose residue on epidermal growth factor (EGF) repeats of Notch receptors [77,78], which participate in the process of cell proliferation, migration, differentiation, and survival [79,80]. The *CYP3A* subfamily represents the largest portion of *CYP* proteins, which play essential roles in the metabolic clearance of wild chemically diverse compounds including environmental pollutants, pesticides, therapeutic drugs, dietary products, and steroids [81,82]. Nesprin-1 has been identified to regulate the cellular functions and diseases [83,84,85,86]. Abnormal cellular functions and subsequent disease pathogenesis may be caused because of an abnormal connection between the nucleus and F-actin through Nesprin-1, leading to insufficient force transmission to the nucleus.

The candidate male-reproduction-related genes from the androgenic gland included Cuticle protein AM1199, Cuticle protein 21, Pro-resilin, and Mucin-19, which were regulated by *PDHE1*. Previous publications have identified the functions of cuticle protein genes in response and adaption to environmental stresses including salinity, predators, and toxins [87,88,89]. Resilin, which is represented in specialized regions of the cuticle in most insects, has been identified to provide low stiffness, high strain, and efficient energy storage [90,91], thus playing important roles in insect flight [92,93], the jumping mechanism in fleas [94,95], and vocalization in cicadas [96]. Mucins are mainly divided into the cell-associated mucin and gel-forming mucin [97]. Many factors can stimulate the secretion of mucin, including toxins, proinflammatory cytokines, and neuropeptides. Thus, the expression of mucins will be regulated by the inflammatory condition [98,99].

In conclusion, *PDHE1* is an energy-metabolism-related gene that has been identified to regulate the process of male reproduction in *M. nipponense*. The genes regulated by *PDHE1* were selected from the testis and androgenic gland of *M. nipponense* through performing transcriptome profiling analysis, and these were predicted to be involved in the regulation of male reproduction in this species. The energy-metabolism-related metabolic pathways were identified as the main enriched metabolic pathways of DEGs in the present study, including pyruvate metabolism, the Citrate cycle (TCA cycle), Glycolysis/Gluconeogenesis, and the Glucagon signaling pathway, indicating that energy metabolism is an essential physiological process for male reproduction in *M. nipponense*. Thus, these energy-metabolism-related pathways and the DEGs from these metabolic pathways may play essential roles in the regulation of male reproduction in this species. Furthermore, four genes were found to be regulated by *PDHE1* in both the testis and androgenic gland, predicting their potential roles in the regulation of male reproduction in *M. nipponense*. The present study identified novel candidate male-reproduction-related genes in *M. nipponense*, providing valuable evidence for studies on the mechanism of male reproduction as well as promoting the establishment of an artificial technique to regulate the process of gonad reproduction in this species.

## Figures and Tables

**Table 1:** The candidate male-reproduction-related genes regulated by *PDHE1*.

Gene	Accession Number	Species	E-Value	Fold Change (*dsPDHE1* vs. *dsGFP*)	
				Testis	Androgenic Gland
Calcification-associated peptide (Cap)	WGF13344.1	*Macrobrachium nipponense*	6.87 × 10^−8^	21.07	−10.38
Chitin binding-like protein (CBP)	ROT74831.1	*Penaeus vannamei*	3.79 × 10^−5^	3.25	−2.53
Ribosomal protein S3 (RPS3)	KAF6343595.1	*Pipistrellus kuhlii*	0.00011	−3.12	−2.78
Uncharacterized protein LOC113829596 (UP)	XP_027238594.1	*Penaeus vannamei*	4.28 × 10^−6^	6.89	6.37
Glucoside xylosyltransferase 2	XP_045611801.1	*Procambarus clarkii*	8.33 × 10^−5^	−21.36	
Skeletal muscle actin 6	QPC96639.1	*Macrobrachium nipponense*	3.61 × 10^−5^	−19.23	
Polycystic kidney disease protein 1	XP_047488675.1	*Penaeus chinensis*	4.12 × 10^−5^	−13.26	
Cytochrome P450 3A30	XP_037798812.1	*Penaeus monodon*	4.11 × 10^−5^	−12.37	
Nesprin-1	XP_045608233.1	*Procambarus clarkii*	5.18 × 10^−5^	−10.39	
Histidine-rich protein DDB_G0274557	XP_047473847.1	*Penaeus chinensis*	6.77 × 10^−9^		−22.65
Cuticle protein AM1199	XP_042888222.1	*Penaeus japonicus*	8.87 × 10^−9^		−22.47
Cuticle protein 21	XP_045617846.1	*Procambarus clarkii*	2.58 × 10^−5^		−21.50
Pro-resilin-like	XP_047479276.1	*Penaeus chinensis*	4.35 × 10^−5^		−19.85
Mucin-19-like	XP_037798457.1	*Penaeus monodon*	0.00012		−17.23

Note: E-value indicates the accuracy of the alignment and annotation in Nr database (*p* < 0.05).

**Table 2:** Primers used in the present study.

Gene	Primer Sequence
*dsPDHE1*-F	TAATACGACTCACTATAGGGGTGCTCTTAGCACTGGAGGC
*dsPDHE1*-R	TAATACGACTCACTATAGGGCCAAGTAGTGGAAGGCAGGA
*PDHE1-*F	TGACCTTAACGGCAACGAGG
*PDHE1-*R	TGACCTTAACGGCAACGAGG
*CAP*-F	TGGAAGCTCGCCGTAGTTTT
*CAP*-R	CTTGACGAAGTGCTGGTTGC
*CBP*-F	CAAGGGCGTCTTCGAGTTCT
*CBP*-R	AAGTTGCCAGGTTCGGGAAT
*RPS3-*F	ATCATGGAGTCTGGCGCAAA
*RPS3-*R	ATACCCAGCACACCTTGACG
*UP-*F	TGTTGGGCGGGAGTTGAAAT
*UP-*R	GTCCTCCTCACCTTGCATCC
*EIF-*F	CATGGATGTACCTGTGGTGAAAC
*EIF-*R	CATGGATGTACCTGTGGTGAAAC

## Data Availability

The raw data of the present study have been submitted to NCBI with the accession number of BioProject PRJNA863726.

## Supplementary Materials

The following supporting information can be downloaded at: https://www.mdpi.com/article/10.3390/ijms25031940/s1.
